# Supplementary material for: Identification of Freezing-Responsive microRNAs and Their Targets in Chinese Jujube by Small RNA and Degradome Sequencing
Source: Int J Mol Sci. 2026 Sep 4;27(17):7912. doi: 10.3390/ijms27177912 (PMC13566890; doi:10.3390/ijms27177912)
Supplement: Supplementary file 1 [file ijms-27-07912-s001.zip › Supplementary Material S2/novel_miR_226.pdf]

Provisional ID	: novel_miR_226
Score total	: 246.5
Score for star read(s)	: 3.9
Score for read counts	: 238.2
Score for mfe	: 2.8
Score for randfold	: 1.6
Score for cons. seed	:
Total read count	: 479
Mature read count	: 393
Loop read count	: 0
Star read count	: 86

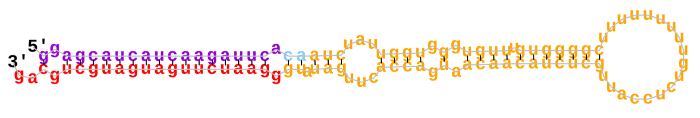

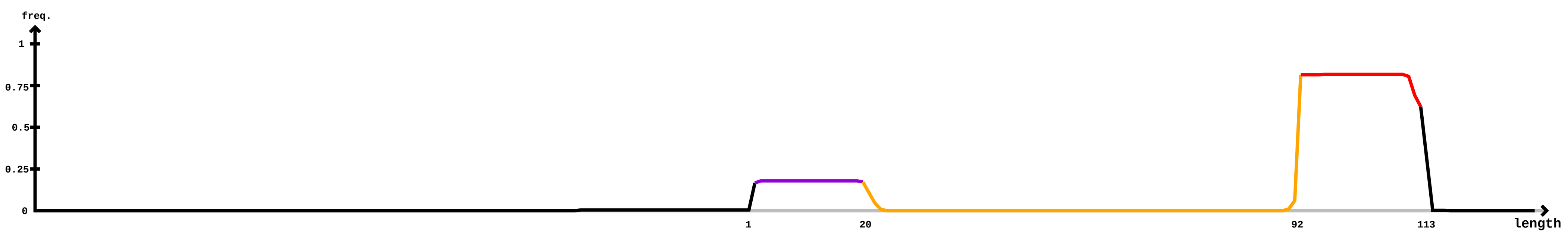

Star Mature

```
5'- aacaaucuucuucuucuccuuuuuaaaaauuuuuaauuuauuuauuuugcauggggauaaugaugguauaaacgaugaggaucuaaaagaagagaagucccagucacuguuugccgauggagcaucaucaagauucacaaucuauuggugggguguuuguggggcuuuuuuuuugucuccauugcucuacaacaaugaccacuugauaugggaaucuugaugaugcugcagcggcaaucaguggcuaaaaa -3' obs
    aacaaucuucuucuucuccuuuuuaaaaauuuuuaauuuauuuauuuugcauggggauaaugaugguauaaacgaugaggaucuaaaagaagagaagucccagucacuguuugccgauggagcaucaucaagauucacaaucuauuggugggguguuuguggggcuuuuuuuuugucuccauugcucuacaacaaugaccacuugauaugggaaucuugaugaugcugcagcggcaaucaguggcuaaaaa exp
    ......(((((.((((((((.....(((((............)))))....)))(((..(.((.((....)).)).)..)))....))))))))))....((((((((.((((((.((.(((((((((((((((((.(((((...((((.(.((((.(((((((.................)))))))))).).))))...))).)).))))))))))))))))).)).)))))).)))))))).....
```

alignment	reads	mm	sample
...aagagaagucccagucacuguuugccgau...	2	0	JCK-2
...gUagcaucaucaagauuc...	1	1	LT-1
...ggagcaucaucaagauuU...	2	1	JCK-2
...ggagcaucaucaagauuca...	2	0	JCK-2
...gUagcaucaucaagauuca...	5	1	JT-3
...gUagcaucaucaagauuca...	1	1	JCK-2
...gUagcaucaucaagauuca...	1	1	JCK-3
...gUagcaucaucaagauuca...	3	1	LT-1
...gUagcaucaucaagauuca...	9	1	LT-3
...gUagcaucaucaagauuca...	12	1	LCK-3
...gUagcaucaucaagauuca...	4	1	JCK-1
...gUagcaucaucaagauuca...	4	1	LT-2
...ggagcaucaucaagauuca...	1	0	LT-3
...gUagcaucaucaagauuca...	5	1	JT-2
...ggagcaucaucaagauucac...	6	0	JCK-2
...gUagcaucaucaagauucac...	1	1	LT-1
...ggagcaucaucaagauucac...	2	0	JCK-3
...ggagcaucaucaagauucac...	1	0	JCK-1
...ggagcaucaucaagauucac...	2	0	LT-1
...gUagcaucaucaagauucac...	2	1	JCK-2
...ggagcaucaucaagauucaca...	1	0	JCK-1
...ggagcaucaucaagauucaca...	3	0	JCK-3
...ggagcaucaucaagauucaca...	5	0	LT-2
...ggagcaucaucaagauucaca...	4	0	JCK-2
...ggagcaucaucaagauucaca...	3	0	LT-3
...gagcaucaucaagauucaca...	1	0	JCK-2
...Uagcaucaucaagauucaca...	1	1	LT-2
...gagcaucaucaagauucacaa...	1	0	JCK-3
...gagcaucaucaagauucacaa...	2	0	JCK-1
...gagcaucaucaagauucacaa...	1	0	JCK-2
...ugggaaucuugaugaugcug...	1	0	LT-2
...ugggaaucuugaugaugcugc...	2	0	JCK-1
...ugggaaucuugaugaugcugc...	1	0	JCK-3

Star — ggagcaucaucaagauuca

Mature — ggaaucuugaugaugcugcag

Reference: aacaaucuucuucuucuccuuuuuaaaaauuuuuaauuuauuuauuuugcauggggauaaugaugguauaaacgaugaggaucuaaaagaagagaagucccagucacuguuugccgauggagcaucaucaagauucacaaucuauuggugggguguuuguggggcuuuuuuuuugucuccauugcucuacaacaaugaccacuugauauagggaaucuugaugaugcugcagcggcaaucaguggcuaaaaa

ugggaaucuugaugaugcugc	1	0	JCK-2
gggaaucuugaugaugcug	1	0	JCK-1
gggaaucuugaugaugcug	2	0	LT-1
gggaaucuugaugaugcugc	1	0	JCK-2
gggaaucuugaugaugcugc	1	0	LT-3
gggaaucuugaugaugcugca	2	0	JCK-2
gggaaucuugaugaugcugca	2	0	LT-2
gggaaucuugaugaugcugca	5	0	JCK-1
gggaaucuugaugaugcugAa	1	1	LT-1
gggaaucuugaugaugcugca	2	0	LT-1
gggaaucuugaugaugcugca	3	0	JCK-3
gggaaucuugaugaugcugcag	1	0	JCK-3
Cggaaucuugaugaugcugcag	1	1	JCK-3
gggaaucuugaugaugcugcag	1	0	JCK-1
Cggaaucuugaugaugcugcag	1	1	JCK-2
ggaaucuugaugaugcug	2	0	LT-3
ggaaucuugaugaugcugc	2	0	JT-2
ggaaucuugaugaugcugc	9	0	JCK-3
ggaaucuugaugaugcugc	7	0	LT-1
ggaaucuugaugaugcugU	1	1	LT-3
ggaaucuugaugaugcugc	8	0	JCK-1
ggaaucuugaugaugcugc	9	0	JCK-2
ggaaucuugaugaugcugc	5	0	LT-2
ggaaucuugaugaugcugc	7	0	JT-3
ggaaucuugaugaugcugca	6	0	JT-3
ggaaucuugaugaugcugca	1	0	LT-3
ggaaucuugaugaugcugca	1	0	JCK-3
ggaaucuugaugaugcugca	5	0	JT-2
ggaaucuugaugaugcugca	5	0	LT-1
ggaaucuugaugaugcugcag	3	0	LT-1
ggaaucuugaugaugcugcNg	1	1	JCK-1
ggaaucuugaugaugcugcag	1	0	JT-3
ggaaucuugaugaugcugcag	121	0	JCK-2
ggaaucuugaugaugcugcag	68	0	JCK-1
ggaaucuugaugaugcugcag	70	0	JCK-3
ggaaucuugaugaugcugcag	1	0	LT-2
ggaaucuugaugaugcugcag	15	0	LT-3
ggaaucuugaugaugcugcag	6	0	LCK-3
ggaaucuugaugaugcugcag	3	0	JT-2
ggaaucuugaugaugcugcagc	1	0	JCK-3
ggaaucuugaugaugcugcagc	1	0	JCK-2
ggaaucuugaugaugcugcagA	2	1	LT-1
ggaaucuugaugaugcugcagA	2	1	JCK-1
ucuugaugaugcugcagcggc	1	0	JCK-3
